# In-Vitro Comparison of Fracture Strength of Endocrowns and Overlays in Endodontically Treated Teeth Manufactured with Monolithic Lithium Disilicate and Zirconia

**DOI:** 10.3390/jfb14080422

**Published:** 2023-08-11

**Authors:** Maya Veselinova, Sofia Diamantopoulou, Chariklia Paximada, Efstratios Papazoglou

**Affiliations:** Department of Operative Dentistry, National and Kapodistrian University of Athens, 11527 Athens, Greece; magiaves@yahoo.gr (M.V.); sophia.diamantopoulou@gmail.com (S.D.); xpaksim@dent.uoa.gr (C.P.)

**Keywords:** endocrowns, overlays, monolithic lithium disilicate, monolithic zirconia

## Abstract

To evaluate the fracture strength and the failure mode of endodontically treated molars restored with monolithic lithium disilicate and zirconia endocrowns and overlays. A total of 48 extracted mandibular molars were endodontically treated, decoronated 2 mm above the cementoenamel junction and divided into four 12-specimen groups. Group ELD: lithium disilicate endocrowns. Group EZ: monolithic zirconia endocrowns. Group OLD: lithium disilicate overlays. Group OZ: monolithic zirconia overlays. Overlays did not extend in the pulp chamber and endocrowns extended in the pulp chamber 2 mm. After adhesive bonding of the restorations, the specimens were subjected to thermocycling (×5000 cycles) and then to fracture resistance testing at lateral static loading (1 mm/min) at a universal testing machine. The failure mode of the specimens was qualitatively evaluated. Differences in means were compared using with t-tests for independent samples or Mann–Whitney test (*p* < 0.05). Weibull distribution analysis was also performed. Group ELD showed significantly higher fracture strength than all other groups (*p* = 0.001), and the highest Weibull modulus. Conclusions: Lithium disilicate endocrowns exhibit higher fracture strength and are more reliable compared to the other types of restorations examined. Endocrowns had more catastrophic failures compared to overlays.

## 1. Introduction

Restoration of endodontically treated teeth remains a subject of debate in the literature and a potential source of confusion for clinicians when deciding on the most appropriate technique and materials. Full-coverage restorations used to be the traditional approach and had high survival rates; however, their placement requires significant tissue removal [1]. The modern approach is shifted towards more conservative solutions: the basic principle is to preserve as much dental tissue as possible using adhesive techniques [2,3,4,5]. Moreover, sound scientific evidence to support the use of full-coverage versus partial-coverage restorations in endodontically treated teeth is still lacking [6,7,8].

Without the need for mechanical retention, which is mandatory for full-coverage restorations, most of the remaining sound tooth can be preserved and receive an indirect partial- coverage restoration. The restoration is bonded to the remaining tooth tissues, with enamel presence and immediate denting sealing technique being very important factors [9]. Moreover, by cusp coverage, extreme flexure of the cusps is avoided, and the occlusal forces are better distributed, resulting in increased tooth fracture resistance [10,11].

Endocrowns are conservative, partial coverage restorations, that are bonded to the internal portion of the pulp chamber and on the cavity margins, thereby resulting in both macro- and micro-mechanical retention, provided by the pulpal walls and adhesive cementation, respectively [12]. In vitro studies show that molars restored with endocrowns resist normal chewing forces without fracture or debonding [13,14,15,16,17]. Survival rates of up to 99% in posterior teeth at 10 years [18] and 90.5% in molars at 12 years [19] have been reported, indicating that this type of restoration could be a long-term alternative approach to the restoration of endodontically treated posterior teeth [12,20,21]. 

It remains unclear whether the extension into pulp chamber offers any advantages, in other words if endocrowns perform better than overlays. No difference was found in fracture strength between endocrowns and overlays made of reinforced composite resin in molars [22,23]. However, when overlays were reinforced with fiberglass, they appeared to have significantly greater fracture resistance than endocrowns with and without fiberglass [23]. Nevertheless, comparison between endocrowns and overlays needs further investigation.

Several materials have been used for endocrown manufacturing: composite resins, ceramic-reinforced composite resins, feldspathic and lithium disilicate ceramics, zirconia-reinforced ceramics, polymer-matrix ceramics, and monolithic zirconia. Fracture resistance of teeth restored with endocrowns from different materials is quite high with small differences between them [9,24,25,26,27,28,29]. Fracture resistance is similar or in some cases even exceed that of full coverage restorations or the intact tooth [13,24,25,28,30,31]. 

Monolithic zirconia is a very widespread material in dentistry due to its relatively low cost and its very good mechanical, physical and optical properties. It does not require etching; therefore, the bonding process can be less sensitive and faster. Its use as a material for endocrowns is relatively new with very limited clinical data available [32,33]. Even the in vitro studies comparing monolithic zirconia endocrowns to other materials are few and inconclusive [34,35,36]. 

The purpose of this in vitro study was to compare the mechanical behavior of endodontically treated teeth restored with endocrowns or overlays fabricated with either monolithic lithium disilicate or monolithic zirconia. The null hypothesis was that there is no difference in fracture strength and failure type between the two restorations design (endocrowns and overlays), and between the two materials, monolithic lithium disilicate and monolithic zirconia.

## 2. Materials and Methods

### 2.1. Cavity Preparation

**Figure 1:**
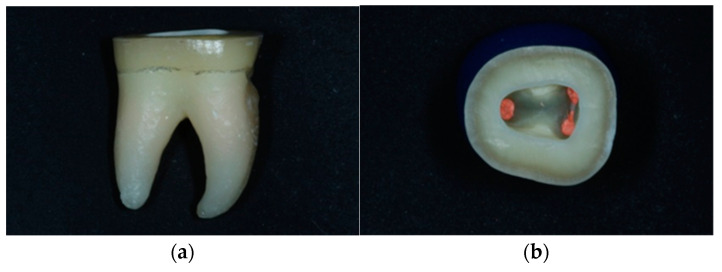
(**a**) Horizontal preparation parallel to the occlusal surface 2 mm above the cementoenamel junction. (**b**) Horizontal preparation with a peripheral range of dental tissues of approximately 3 mm. Maintenance of the morphology of the pulp chamber. Smooth and divergent walls 8–10°.

For the purposes of the present study, 48 extracted, caries-free mandibular human molars with complete root formation were selected. All subjects gave their informed consent for use of their extracted teeth for the purposes the study. The study was conducted in accordance with the Declaration of Helsinki, and the protocol was approved by the Research Ethics Committee of the Dental School (393A/29.11.2018). The teeth had similar mesiodistal and buccolingual dimensions and were stored in 0.1% thymol solution at room temperature. All teeth were prepared for endodontic treatment, with diamond burs in high-speed handpiece and tungsten carbide burs in low-speed handpiece. Root canals were mechanically shaped with NiTi instruments (Protaper Universal Rotary; Dentsply, Johnson City, TN, USA) and a low-speed handpiece (Endomate, NSK, Eschborne, Germany) with continuous 5% sodium hypochlorite irrigation. Root canal obturation was performed with warm gutta-percha points (Gutta-percha Points, Dentsply, USA) and epoxy resin filling (AH Plus, Dentsply, USA) combined with a vertical compaction technique (System B, Sybron Dental, Orange, CA, USA). The gutta-percha was cut 1 mm below the orifices of the root canals. Cusps were cut parallel to the occlusal surface 2 mm above the cementoenamel junction (Figure 1a). Margin preparation was performed horizontally with a width of about 3 mm (Figure 1b). The morphology of the pulp chamber was preserved, internal angles were rounded, and recesses were eliminated. The walls of the pulp cavity were divergent 8–10° (Figure 1b).

Root canal orifices were covered with low viscosity composite resin (Tetric EvoFlow, Ivoclar Vivadent, Schaan, Liechtenstein), after application of bonding agent (Adhese Vivapen, Ivoclar Vivadent, Schaan, Liechtenstein) and polymerization for 20 s, with a LED lamp (Bluephase Style, Ivoclar Vivadent, Schaan, Liechtenstein, 1100 mW/cm^2^). Teeth were then randomly divided into two groups, half to be restored with endocrowns and half with overlays.

**Figure 2:**
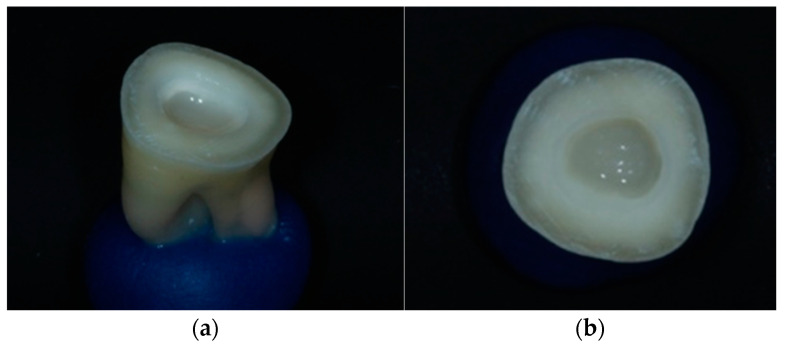
(**a**) Pulpal chamber filling with low viscosity composite resin. (**b**) Pulp chamber extension of 2 mm.

**Figure 3:**
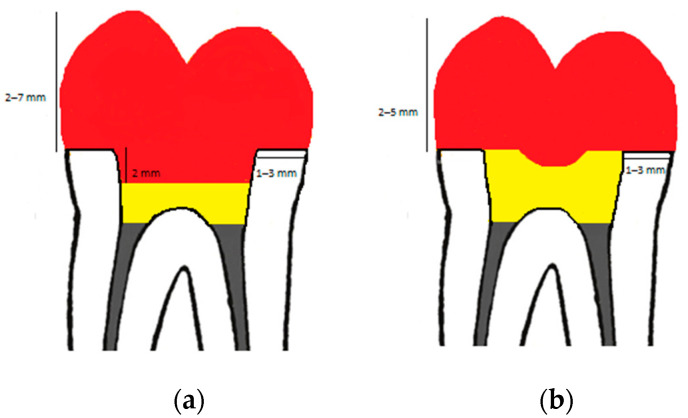
Tooth preparation design of endocrown (**a**) and overlay (**b**) with dimensions. Red color: restoration (endocrown/overlay), yellow color: resin composite, grey color: guttapercha.

In the endocrowns group, the pulp chamber floor was filled with low-viscosity composite resin until the intrapulpal extension of the final restoration was 2 mm [37] (Figure 2 and Figure 3).

**Figure 4:**
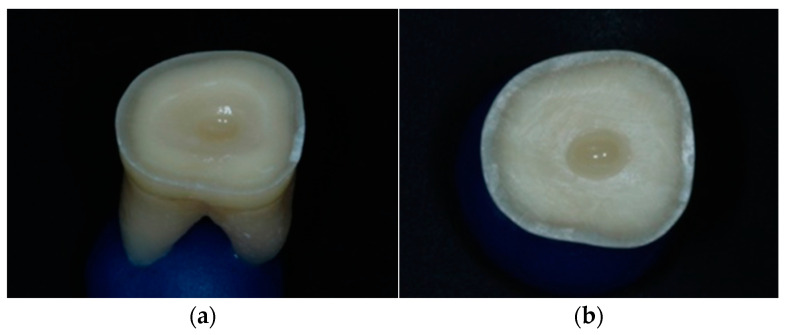
(**a**) Filling of the entire pulp chamber with low-viscosity composite resin. (**b**) The circular recess in the resin mass for proper seating of the restoration.

In the overlays group, the entire pulp chamber was filled with low-viscosity composite resin and a circular recess of 2 mm diameter was formed in the resin mass to help seating of the overlay in the correct position (Figure 4). 

Each group was randomly divided into two equal subgroups, half to be restored with monolithic zirconia and half with monolithic lithium disilicate.

Digital impressions of the prepared teeth were taken with an intraoral scanner (TRIOS^®^ 4, 3Shape, Copenhagen, Denmark).

After the impression, teeth were kept in saline solution until the restorations were fabricated and bonded, without any temporary filling material.

### 2.2. Restoration Fabrication

A total of 24 restorations (12 endocrowns and 12 overlays) were fabricated from monolithic lithium disilicate (IPS e.max CAD, Ivoclar Vivadent, Schaan, Liechtenstein) and 24 restorations (12 endocrowns and 12 overlays) from monolithic zirconia (IPS e.max Zir CAD Multi, Ivoclar Vivadent, Schaan, Liechtenstein). They were designed with exocad software (Exocad GmbH, Darmstadt, Germany) and manufactured with Roland DWX-52DC cutting machine (Roland DG Corporation, Shizuoka, Japan) using MillBox software (DGShape, Shizuoka-ken, Japan). The occlusal restoration thickness was 5 mm.

### 2.3. Bonding Procedure

The marginal fit of the final restorations was checked with an explorer. The teeth were air-abraded with 50-μm Al_2_O_3_ particles (Microetcher IIA, Danville, Carlsbad, CA, USA). Enamel was etched with 37% orthophosphoric acid (Total Etch, Ivoclar Vivadent, Schaan, Liechtenstein) for 30 s, rinsed and dried. Single-use activator (Monobond Plus, Ivoclar Vivadent, Schaan, Liechtenstein) was applied for 60 s to the composite resin covering part or all the pulp chamber of the tooth. Finally, bonding agent (Adhsese Universal, Ivoclar Vivadent, Schaan, Liechtenstein) was applied to the entire surface of the tooth for 20 s, dried and light-cured for 10 s, according to the manufacturer’s instructions.

Lithium disilicate restorations were cleaned with Ivoclean paste (Ivoclar-Vivadent, Schaan, Liechtenstein) for 20 s, rinsed and air-dried. They were etched with 4.9% hydrofluoric acid (IPS ceramic etch, Ivoclar-Vivadent) for 20 s and rinsed with water for 30 s. Afterwards, they were placed in an ultrasonic bath for 1 min and air-dried. Finally, a single-use activator (Monobond Plus, Ivoclar-Vivadent, Schaan, Liechtenstein) was applied for 60 s and air-dried.

Zirconia restorations were cleaned with Ivoclean paste (Ivoclar-Vivadent, Schaan, Liechtenstein) for 20 s, rinsed and air-dried. They were air-abraded with 50-μm Al_2_O_3_ particles (Microetcher IIA, Danville, USA), rinsed, placed in an ultrasonic bath for 1 min and air-dried. Finally, a single-use activator (Monobond Plus, Ivoclar-Vivadent, Schaan, Liechtenstein) was applied for 60 s and air-dried.

Dual-cure luting resin cement (Variolink Esthetic DC, Ivoclar Vivadent, Schaan, Liechtenstein) was used for bonding of all the restorations, according to the manufacturer’s instructions. After seating of the restorations, the excess cement was polymerized for 2 s so that it could be easily removed, an oxygen inhibition gel (Liquid Strip, Ivoclar-Vivadent, Schaan, Liechtenstein) was applied to the margins and the final photopolymerization was performed for 20 s on each surface with the LED lamp. The gel was rinsed, the excess cement was removed with a No15 scalpel, and the surfaces were finished and polished with 2-step, flame-shaped diamond rubber polishers (Optragloss, Ivoclar Vivadent, Schaan, Liechtenstein) under continuous water irrigation. 

In total, there were 4 groups with 12 specimens each:

(1) Monolithic lithium disilicate endocrowns (ELD);
(2) Monolithic lithium disilicate overlays (OLD);
(3) Monolithic zirconia endocrowns (EZ);
(4) Monolithic zirconia overlays (OZ).

### 2.4. Aging and Fracture Strength Test

After bonding, specimens were placed in saline solution for 24 h at room temperature. They were then subjected to 5000 cycles of thermocycling in a water bath at temperature 5 °C to 55 °C, with a dwell time at each temperature of 30 s.

Teeth were embedded in self-curing acrylic resin (VersoCit-2 Kit, Struers) 1 mm below the cementoenamel junction in metallic cylinders with an inner diameter of 1.3 cm and an outer diameter of 1.6 cm, as shown below.

**Figure 5:**
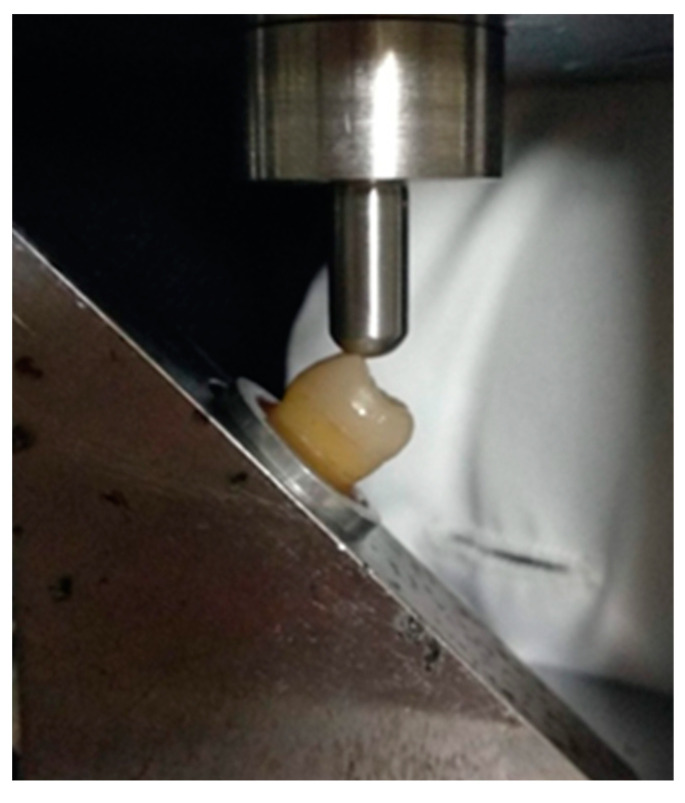
Specimen mounted on the universal testing machine. A 6 mm diameter stainless-steel round ball applies a compressive force at an angle of 45° to the long axis of the tooth on the buccal cusps.

Finally, specimens were subjected to fracture strength test using a universal testing machine (Tensometer 10, Monsanto, Swindon, UK). A 6 mm diameter stainless-steel ball at crosshead speed of 1 mm/min, applied a compressive force at an angle of 45° to the long axis of the tooth on the buccal cusps of each specimen (Figure 5). Load was applied until the restoration was debonded or fractured. At that time, the loading force in Newtons, which expresses the fracture strength of each specimen, was recorded.

To overcome the limitations of brittle fracture of materials and the associated problems of wide dispersion of shear values, Weibull analysis was used which expresses the probability of failure with the corresponding confidence limits (95%). This method has been shown to be the best for processing fracture test results [38].

Finally, all fractured surfaces were analyzed using an optical microscope (DM 4000B, Leica Microsystems) in reflection mode under 75× magnification to identify failure modes. The type of failure was classified as cohesive type in the restoration material (type I), adhesive type between the restoration material and dentin (type II), cohesive in enamel/dentin (above the CEJ) (type III), IV: fracture extending to root (below the CEJ) (type IV). 

### 2.5. Statistical Analysis

Distributions of the data were tested with the Shapiro–Wilk test. Descriptive statistics were calculated including means and standard deviations for normally distributed data and medians with interquartile ranges for skewed data. Differences between zirconia-lithium disilicate (overlays or endocrowns), and differences between endocrowns-overlays (zirconia or lithium disilicate) were assessed with *t*-tests for independent samples or Mann–Whitney test, respectively. A chi-square statistical test was performed, with criteria the material of the restoration (ELD-EZ and OLD-OZ) and the extension in the pulp chamber (ELD-OLD and EZ-OZ). The fracture strength data were analyzed by Weibull analysis. The shape or modulus parameter-β (defines the variability of the results, by expressing the size distribution of the flaws), the scale or B63.2 parameter-σ0, (defines the characteristic life, by indicating the strength value for which the 63.2% of the sample size was debonded) and the strength at 10% failure probability (σ0.1) of the Weibull distributions were calculated. *t*-test, Mann–Whitney test and chi-square test were performed by SigmaStat software (SigmaPlot v.12.5, Systat Software Inc, San Jose, CA, USA). For the Weibull analysis, the OriginLab software (v.9.1 SRO, Northampton, MA, USA) was used and for graph the softoware Develpe (v. 3.12, Velp, The Netherlands, for Weibull analysis). For all cases, a 95% confidence level was selected (α = 0.05).

## 3. Results

The mean fracture strength values and standard deviations of the groups ELD, OLD, and ELD, EZ expressed in Newton are presented in Table 1 and Table 2. The medians with interquartile ranges of the groups EZ, OZ and OLD, OZ expressed in Newton are presented in Table 3 and Table 4.

A chi-square statistical test was performed, with criteria the material of the restoration (ELD-EZ and OLD-OZ) and the pulp chamber extension (ELD-OLD and EZ-OZ) with a confidence level of 95% (α = 0.05). For the material, no statistically significant difference in failure type was found between groups ELD-EZ (*p* = 1) and OLD-OZR (*p* = 0.08). For the extension in the pulp chamber, a statistically significant difference was found between groups ELD-OLD, but not between groups EZ-OZ.

**Figure 6:**
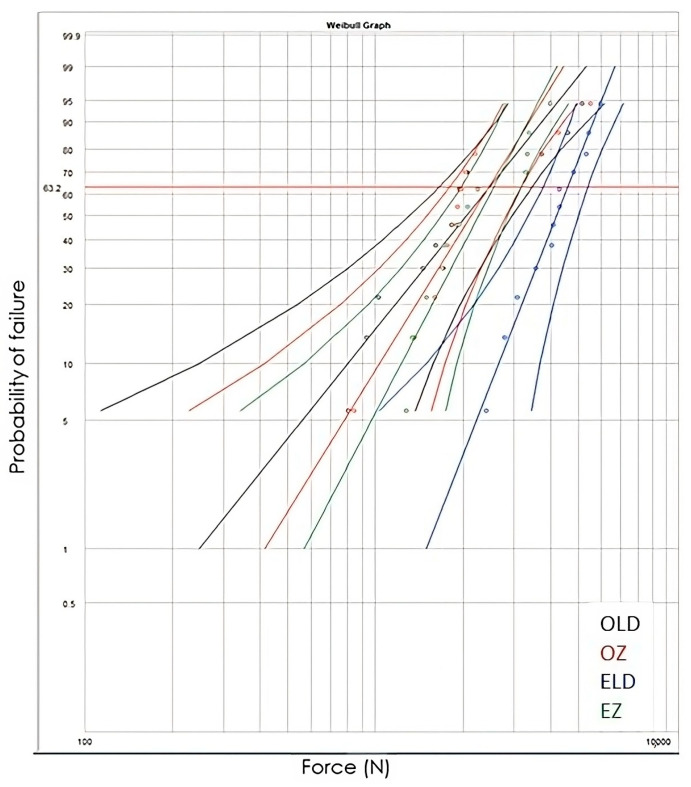
The Weibull diagram for the four groups tested.

The results from the Weibull analysis are presented in Table 5 and the Weibull diagram is presented in Figure 6. The Weibull modulus (m), which defines the variability of the results, was determined. The scale parameter σ_0_, which shows the characteristic strength or life, was also determined, indicating the strength value at which 63.2% of the sample size failed. According to Table 2, the most reliable and strong restoration is the lithium disilicate endocrown, with a statistically significant difference from the others. For the ELD group the strength value was significantly higher compared to the other groups. For the ELD group the breaking load value (4583.6) was higher than the other groups to a statistically significant degree, OLD (2479.1), EZ (2558), and OZ (2459.4). The Weibull distribution graph is depicted in Figure 6. A high modulus, or steep slope, is associated with a narrow strength distribution. This is considered an advantage for a material, as it implies predictability. A material with high Weibull moduli is less probable to fail at a stress much lower than a mean value.

**Figure 7:**
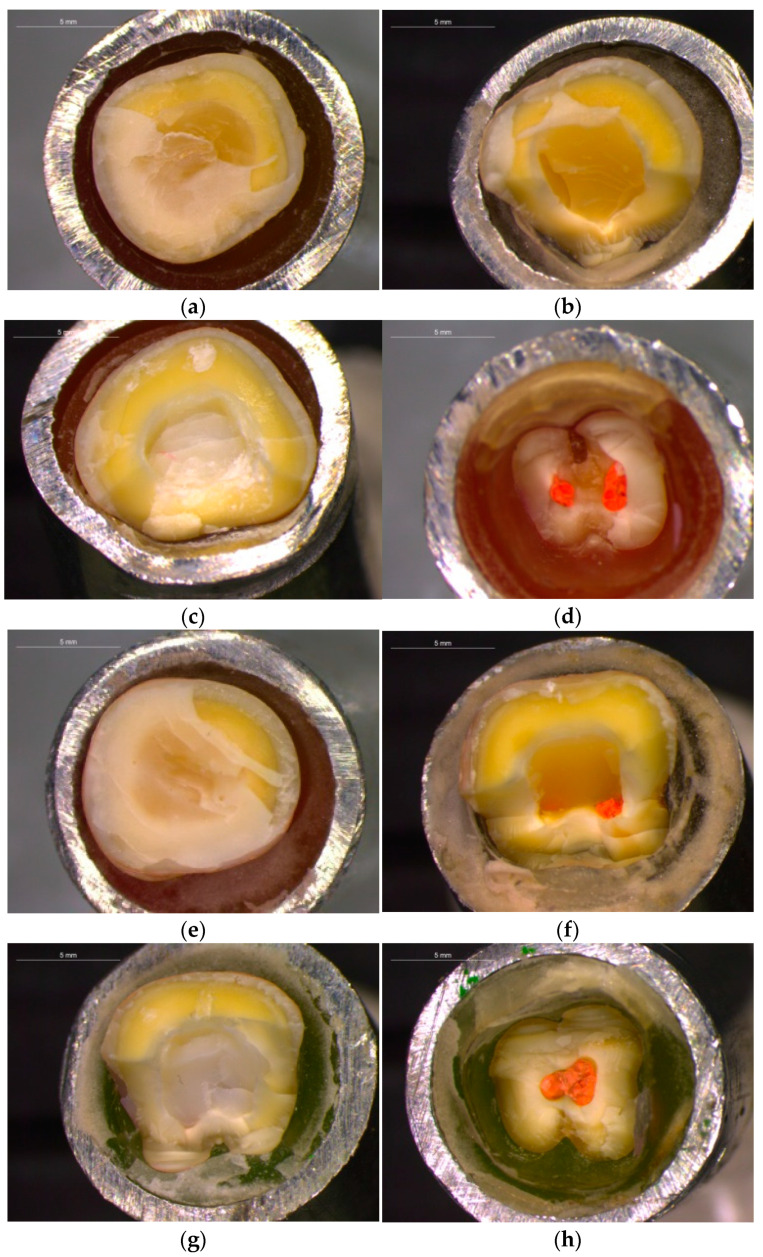
Representative failure types of restorations: (**a**) type II group OLD, (**b**) type IV group OLD, (**c**) type III group ELD, (**d**) type IV group ELD, (**e**) type II group OZ, (**f**) type IV group O, (**g**) type IV group EZ, (**h**) type IV group EZ.

The total number of each type of failure per group of samples is presented in Table 6. Failures were categorized in four types. I. Cohesive in the restoration material; II. Adhesive between the restoration material and dentin; III. Cohesive in enamel/dentin (above the CEJ); IV. Facture extending to root (below the CEJ). Photos of each fracture type per group are presented in Figure 7a–h. No statistically significant difference in failure type was found between the groups ELD-EZ and OLD-OZ. Statistically significant differences were found between groups ELD-OLD and EZ-OZ.

## 4. Discussion

In the present study, the effect of the material (lithium disilicate or zirconia) and the extension or not in the pulp chamber (endocrown or overlay) of bonded restorations in endodontically treated mandibular molars was evaluated. Lithium disilicate endocrowns (group ELD) had statistically higher fracture strength compared to the other groups. Weibull analysis also indicated that lithium disilicate endocrown was the most reliable and strong restoration. In general, the type of failure of endocrowns was predominately type IV, which was more destructive to the teeth compared to overlays. Therefore, the null hypothesis was partially rejected.

In the current research, endocrowns exhibited higher fracture strength compared to overlays when the material of choice was lithium disilicate. Extending the restoration into the pulp chamber increases the bonding surface as well as the macromechanical retention, preventing its debonding under lateral forces. Lateral movements are more detrimental than axial ones for adhesive interfaces [25]. This can possibly explain why lithium disilicate endocrowns exhibited greater fracture strength than lithium disilicate overlays, which rely solely on adhesion. In a previous study conducted on premolars, authors concluded that the use of flat overlays with only adhesive retention is discouraged, since lithium disilicate endocrowns outperformed lithium disilicate overlays [39]. In another study conducted on molars, similar results were reported [40]. Nevertheless, most overlay failures were debondings without fracture of the tooth. On the other hand, when endocrowns failed, this was more destructive for the tooth as it appeared as a fracture below the cementoenamel junction [40]. This is consistent with this study’s findings.

When monolithic zirconia was examined, there was no difference between endocrowns and overlays concerning the fracture strength. However, there were differences in the failure type. All monolithic zirconia endocrowns exhibited catastrophic failures as they fractured below the cementoenamel junction. On the other hand, half of the monolithic zirconia overlays debonded without tooth fracture, while the other half debonded and teeth fractured below the cementoenamel junction.

In clinical practice, the tooth is evaluated after the failure occurs, to see whether the residual structure is repairable or not. Therefore, understanding the fracture pattern and the type of stress distribution are equally crucial to considering the fracture load. While the lithium disilicate overlay group primarily experienced repairable fractures, the zirconia endocrown group had catastrophic failures that might reach 100%. This is consistent with earlier research’ findings that zirconia endocrowns had a high rate of catastrophic failure [34,35,36,41,42,43]. 

Lithium disilicate endocrowns exhibited higher fracture strength than zirconia endocrowns. This agrees with the findings of a previous study [44]. This difference can probably be attributed to the higher modulus of elasticity of zirconia, which results in tooth fractures under lower forces and in a shorter time than those of lithium disilicate endocrowns. In a previous study finite element analysis showed that for lithium disilicate endocrowns, Von Misses stresses under lateral forces are concentrated in the enamel, the dentin, and the restoration itself [45]. On the other hand, for zirconia endocrowns, stresses are higher in the dentin and lower in the enamel, restoration’s center, and luting cement. Lower stress concentration in the cement layer means that adhesion is beneficial for the restoration. The endocrowns made of greater elastic modulus ceramics exhibit higher Von Misses stress [46], which is transferred to the remaining tooth structure, increasing the risk of tooth fracture. The CAD/CAM lithium disilicate ceramic used in the present study has a modulus of elasticity of 95 GPa [47]. The second material used, 4Y-TZP, Yttria stabilized-Tetragonal Zirconia Polycrystal has a modulus of elasticity of >200 GPa [48]. The deformation of the zirconia restoration under load application is significantly smaller compared to that of the lithium disilicate restoration under the same conditions [45]. The importance of the elastic modulus of both the materials and the dentin has a significant impact on how the ceramics fail during the loading test. In contrast to stiff materials with higher elasticity moduli than dentin, which led to stress accumulation and catastrophic failures, the stresses found in materials with closer elasticity moduli to dentin were dispersed. 

In some earlier studies, monolithic zirconia outperformed other ceramics, notably lithium disilicate, in terms of mechanical performance [35,49]. The variations may be explained by the different conditions of each experiment. In one study [35], the force was applied perpendicular to the occlusal surface of the teeth, while in the present study the compressive force had at an angle of 45°. In another study [49], central incisors were used. In both studies monolithic zirconia showed a higher rate of catastrophic tooth structure failure [35,49], which agrees with the present one.

For males and females, respectively, the mean axial masticatory forces in humans range from about 600 to 900 N [50,51,52]. There is a lack of information about the magnitude forces on the jaw or teeth from the lateral direction during oral function in humans, but based on a theoretical and validated model it is assumed that these forces are in the range of 200 N [53,54]. Based on other research, the occlusal force in the molars is typically between 222 to 445 N and can reach up to 800 N during clenching [55,56]. The measured values under oblique stress at the time of fracture in the current investigation were much higher than these values. Nevertheless, direct comparisons with the results of other studies cannot be performed since it is impossible to have all the parameters identical. Variations in the selected materials and teeth, the method of fatigue or aging, the bonding techniques, and the test methods contribute to differences in the results obtained.

In the overlay’s groups, there were no differences in fracture strength between the two restorative materials. Since the fracture strength of overlays relies solely on adhesion, the results of the present study indicate that bonding of monolithic zirconia to dental tissues was comparable to lithium disilicate. Polycrystalline zirconia ceramics are not etchable like glass-ceramics, therefore some method of priming the bond surface is necessary to achieve a satisfactory bond. In the present study, sandblasting with 50 μm Al_2_O_3_ was used, during which surface cleaning, increase in surface roughness, increase in wettability, and micromechanical retention are achieved [57]. Sandblasting must be followed by a primer capable of improving adhesion such as self-adhesive cements and primers containing acid phosphate monomers [57]. 

Molars, and not premolars, were used for the experiment, as endocrowns were initially suggested for molars. For quite a long-time, premolars were not considered good candidates for endocrowns. Endocrowns on premolar teeth may not be appropriate because of the smaller pulp chamber space’s reduced bonding surface area, according to one study [58]. In 2020, Govare and Contrepois advocated endocrowns as a trustworthy alternative for post-retained restorations in molars and suggested that additional clinical research is needed before using endocrowns on premolars [59]. Nevertheless, premolars can be candidates for endocrowns, according to a recent systematic review and meta-analysis that found no difference in the failure rate between endocrowns on molars and premolars [59]. Lower molars were used in the present study as it was easier to collect teeth with similar dimensions and anatomy in contrast to upper molars.

Regarding the thickness of the restoration above the cusps, it was chosen to be 5 mm, even though there are no clear guidelines on this topic. It is advisable, however, not to exceed this thickness, as it was found that stresses increase [14]. Restoration thickness between 3–7 mm has been reported [60,61]. Moreover, one main indication of endocrowns is teeth with short clinical crowns and a limited interocclusal space.

As far as the extension of the endocrown into the pulp chamber is concerned, results are conflicting and range from 2 to 5 mm. In a previous study no significant difference in fracture resistance was observed between endocrowns of 2 or 4-mm [37]. For the present study, a 2 mm extension in the pulp chamber was chosen. In a pilot study, it was observed that a deeper chamber extension led to more catastrophic failures.

For artificial aging of the samples, the method of thermocycling was used, which represents laboratory hydrothermal aging. Temperatures, residence times and cycles of this process vary in different laboratory studies [62]. Hydrothermal aging is a topic with conflicting reviews in the literature. In some studies, it does not seem to affect adhesion [63,64] while in others it appears to do so [65,66]. No other aging method was used, such as long-term storage in a humid environment at 37 °C or mechanical loading in a chewing simulator. This probably explains the high fracture strength values compared to other studies. Additional aging processes are expected to cause further weakening of the bond with possible consequent reduction in fracture strength.

During the fracture test, the force was applied at a 45° angle to the buccal cusps (functional cusps of lower molars) producing shear stresses that are common in the oral environment during mastication and are more destructive to bonding interfaces than stresses applied perpendicular to the occlusal surface. Under the influence of lateral forces, one would expect that the extension of the endocrown in the pulp chamber will enhance the macromechanical retention, as opposed to the flat overlay, as was performed in the lithium disilicate group. In other studies, the force is applied perpendicular to the occlusal surface simulating a state of ideal occlusion in the molar region and is perhaps overestimating the results [23].

As with all in vitro studies, the present one has some limitations in terms of simulating clinical conditions. Although fracture resistance was considered, the biomechanical properties of the periodontium were not included. An artificial periodontal ligament was not used in the present study, because the materials used for this purpose show degradation during the experimental process. During testing, this can cause the tooth to shift as a result. Previous research has demonstrated that periodontal ligament simulation could alter fracture strength outcomes and failure modes favorably by acting as a shock absorber [67,68]. Moreover, the final thickness of this artificial ligament cannot be easily standardized [67]. One more limitation of the present study is that static load was applied while forces produced intraorally are different in their magnitude, frequency, direction, and speed of application. Furthermore, even though thermocycling was performed to simulate conditions in the oral environment, it was limited to 5000 cycles. Finally, this study did not use artificial saliva to imitate the intra-oral conditions. 

## 5. Conclusions

Within the limitations of the present study, the following conclusions emerge: 

Lithium disilicate endocrowns exhibit higher fracture strength and are more reliable compared to the other types of restorations examined. 

Endocrowns had more catastrophic failures compared to overlays.

The results of the present study could be used as a basis for future research. Randomized control clinical trials are needed to further investigate the topic.

## Figures and Tables

**Table 1:** Mean and standard deviations of fracture strength values of the groups ELD, OLD given in Newton. Different letters indicate statistically significant difference (*p* < 0.001).

Group	Mean Value (SD)
ELD	4169.00 (1090.534) ^a^
OLD	2254.75 (1442.6) ^b^

**Table 2:** Mean and standard deviations of fracture strength values of the groups ELD, EZ given in Newton. Different letters indicate statistically significant difference (*p* < 0.001).

Group	Mean Value (SD)
ELD	4169.00 (1090.534) ^a^
EZ	2312.25 (928.48) ^b^

**Table 3:** Μedians with interquartile ranges of fracture strength values of the groups EZ, OZ given in Newton. Different letters indicate statistically significant difference (*p* < 0.001).

Group	Median Value	25th Percentile	75th Percentile
EZ	2007 ^a^	1545.25	3309.5
OZ	1904.5 ^a^	1631.25	2168

**Table 4:** Μedians with interquartile ranges of fracture strength values of the groups OLD, OZ given in Newton. Different letters indicate statistically significant difference (*p* < 0.001).

Group	Median Value	25th Percentile	75th Percentile
OLD	1873.5 ^a^	1134.25	3315
OZ	1904.5 ^a^	1631.25	2168

**Table 5:** The Weibull analysis for the four groups tested. Different letters show statistically significant difference.

Group	m	σ_0_ (N)	σ_0_ 95% CI	r^2^	Significance (σ_0_, *p* < 0.05)
OLD	2	2479.1	1643–3489	0.89	^b^
OZ	2.6	2459.4	1803.6–3178	0.81	^b^
ELD	4.1	4583.6	3804.7–5.452.7	0.98	^a^
EZ	3.1	2558	1993.3–3157.1	0.88	^b^

**Table 6:** Total number of each type of failure per group.

	N	Type Ι	Type ΙΙ	Type ΙΙΙ	Type IV
ELD	12		-	2	10
OLD	12	-	9	-	3
EZ	12	-	-	-	12
OZ	12	-	6	-	6

## Data Availability

Data Availability Upon Request.
